# A Dual-Padded, Protrusion-Incorporated, Ring-Type Sensor for the Measurement of Food Mass and Intake

**DOI:** 10.3390/s20195623

**Published:** 2020-10-01

**Authors:** Wonki Hong, Jungmin Lee, Won Gu Lee

**Affiliations:** Department of Mechanical Engineering, Kyung Hee University, Yongin 17104, Korea; wk.hong@khu.ac.kr (W.H.); mudoosan@khu.ac.kr (J.L.)

**Keywords:** dietary monitoring, healthcare, ring-type biosensor, personalized digital medicine

## Abstract

Dietary monitoring is vital in healthcare because knowing food mass and intake (FMI) plays an essential role in revitalizing a person’s health and physical condition. In this study, we report the development of a highly sensitive ring-type biosensor for the detection of FMI for dietary monitoring. To identify lightweight food on a spoon, we enhance the sensing system’s sensitivity with three components: (1) a first-class lever mechanism, (2) a dual pad sensor, and (3) a force focusing structure using a ring surface having protrusions. As a result, we confirmed that, as the food arm’s length increases, the force detected at the sensor is amplified by the first-class lever mechanism. Moreover, we obtained 1.88 and 1.71 times amplification using the dual pad sensor and the force focusing structure, respectively. Furthermore, the ring-type biosensor showed significant potential as a diagnostic indicator because the ring sensor signal was linearly proportional to the food mass delivered in a spoon, with *R*^2^ = 0.988, and an average *F*_1_ score of 0.973. Therefore, we believe that this approach is potentially beneficial for developing a dietary monitoring platform to support the prevention of obesity, which causes several adult diseases, and to keep the FMI data collection process automated in a quantitative, network-controlled manner.

## 1. Introduction

The intake amount of food is a critical factor in determining the state of the body’s health because overeating can cause obesity, diabetes, and high blood pressure [1,2,3]. Patients who die from diseases such as cardiovascular disease, diabetes, and cancer account for more than 70% of deaths worldwide. The WHO reports that obesity due to an unhealthy diet and lack of physical activity is one of the leading causes of death [4]. In particular, obesity in children leads to obesity in adults and causes hyperinsulinemia, hyperlipidemia, glucose intolerance, and decreased growth hormone [5]. Moreover, the increase in social costs due to obesity is an economic burden for every country. Therefore, dietary monitoring is necessary for societal, economic and public health reasons. The need for food mass and intake (FMI) information has been increasing because of the knowledge that FMI plays an essential role in revitalizing a person’s health and physical condition.

A wearable type of sensing system is required to detect and analyze intake in real-time throughout the day. In previous research, devices such as a mouthpiece, a skin patch, a wristband, a neckband, a microphone, a camera, and smart glasses were used. Among the previous studies, there was a study to detect food intake patterns using an accelerometer mounted on a mouthpiece [6]. There was also a dietary monitoring method using a skin attachment sensor [7]. Moreover, monitoring FMI has been analyzed using an attachment on a tooth. Although the attachment had an ultra-thin design, it still gave the user a negative perception due to the electrical current flowing in the mouth [8]. Additionally, rejection of the attachment by the body is also a problem to be solved. Separately, there is an indirect dietary monitoring method that measures the freshness of food by the detection of bacteria in tuna broth [9]. Dietary monitoring has also been attempted by sound discrimination using a microphone in a headset. In this case, the reduction of accuracy caused by ambient noise can be an issue [10,11]. In a different example, a sensing method was developed that monitored the swallowing motion using a piezoelectric element or an EGG (electroglottography) sensor in the neckband. This sensor approach focused on only intake pattern, limiting the use of the actual dietary monitoring [12,13].

Dietary monitoring may also be performed by using a smartphone camera to detect the food volume and pattern of intake. However, there is the inconvenience of carrying a camera and the requirement of taking an accurate picture according to instructions in a manual [14,15,16,17,18,19,20]. The eating pattern can also be detected through a wrist-type inertial sensor, such as a watch [21]. There is also a method for detecting the movement of facial muscles, including the oral cavity, with a piezoelectric sensor attached to the side of eyeglasses. However, that system has a problem in that it is difficult to identify the correct pattern because the force recognized by the sensor is insensitive. There is also an issue that the volume of the glasses at the temple increase [22,23]. In summary, the existing dietary monitoring studies had limitations in accurately measuring FMI, which is the core of dietary monitoring. In addition, there was an issue of users not wanting to wear the sensors due to the non-compact form factor.

In this study, we present a new dietary monitoring method using a compact, ring-type sensor on the hands, the final body part involved in eating prior to intake. We develop a highly sensitive finger sensing system using a ring-type flexible force sensor, with data connectivity via Bluetooth. We introduce three factors to enhance the sensitivity of the sensing system for detecting lightweight food. We validate our sensing system via experiments focused on the usability and accuracy of the device. Our results indicate that a wireless, ring-type finger sensing system can measure FMI with high sensitivity and accuracy for dietary monitoring.

## 2. Materials and Methods

### 2.1. Design and Fabrication of the Ring Sensing System

**Figure 1:**
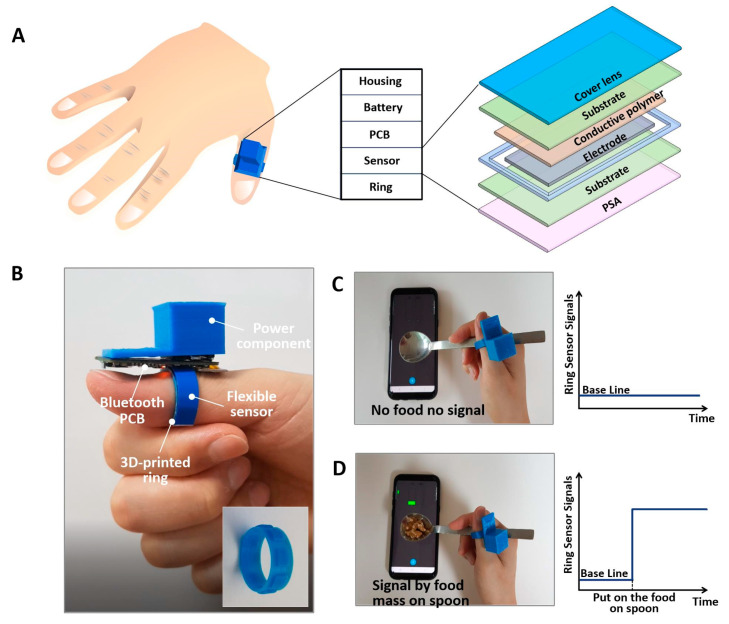
Schematic illustration and photograph of the ring-type biosensor for dietary monitoring via a smartphone. (**A**) Composition layer of ring sensing system: ring, sensor, printed circuit board, battery and housing, and flexible sensor: cover lens, substrate, conductive polymer, electrode, substrate, and pressure-sensitive adhesive. (**B**) Ring-type biosensing system mounted on the thumb and component unit: flexible tactile sensor, 3D-printed ring, power component and circuit board for wireless communication and battery. (**C**) Establishment of a baseline signal for a spoon with no food mass, taken using filtering. (**D**) Signal generated by food mass on the spoon.

The composition layers of the ring sensing system and the flexible sensor are shown in Figure 1A. The layers of the sensing system start from the bottom, consisting of the ring, and include a flexible sensor, a printed circuit board, a battery in a holder, and the housing. The flexible sensor is composed of a cover lens, a polyethylene terephthalate (PET) substrate, a conductive polymer, an electrode, a substrate, and a pressure-sensitive adhesive (PSA). The layer on the top of the electrodes is a high-resistance material in which conductive particles are dispersed in a polymer such as polycarbonate (PC) or poly (methyl methacrylate)(PMMA), and pressure is detected by the contact area’s change with the electrode according to the applied force. The cover lens protects the sensor. The electrodes and conductive polymers are deposited and patterned with a screen printing process.

As shown in Figure 1B, the ring sensor is placed on the bottom of the thumb, the typical contact point when holding a spoon. Here, the ring sensor uses a flexible force-sensing resistor, which detects the resistance change caused by the contact area’s change when pressure is applied. We used a ring apparatus with a unique pattern that can focus the pressure from the spoon contact. The structure is made from polylactic acid and is fabricated using a 3D-printed, fused deposition method. The flexible force sensor is then placed on the structure. The driving voltage, 3 V, is applied to the sensor through two coin LR44 batteries in the battery holder layer, and the system communicates wirelessly with a smartphone via a field-programmable gate array (FPGA) board with a Bluetooth module. We used the compact module, Steval stlcs01v1 by STMicronics(Geneva, Switzerland), for Bluetooth low energy connectivity and pressure sensing. For the smartphone operating system, we used Android version 9(Mountain View, CA, USA). Finally, we confirmed the signal of no spoon mass by taking a new baseline using filtering, and then monitored the signal generated by food mass on the spoon using the wireless system, as shown in Figure 1C,D.

### 2.2. Principle and Theory

**Figure 2:**
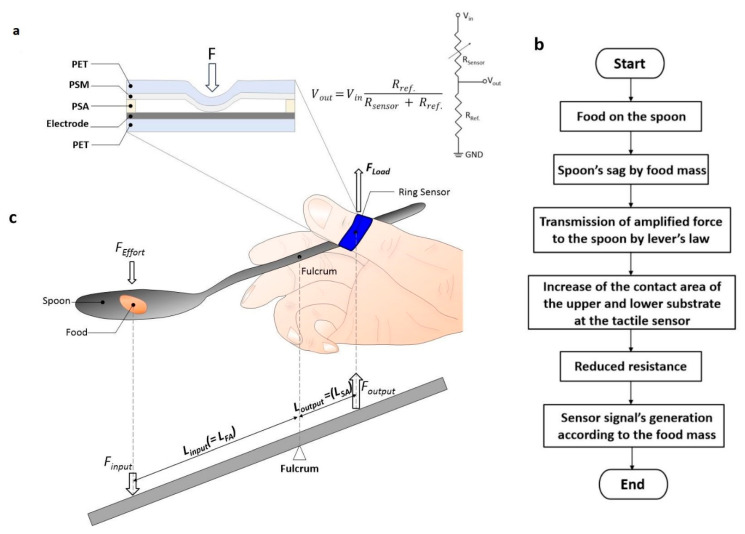
Schematic illustration of the ring-type sensing system’s principle. (**a**) Schematic illustration of the force-sensitive layers of the ring sensor with voltage in-and-out relation. (**b**) Flow chart of the operation mechanism. (**c**) Schematic diagram of the ring-type biosensor using the first-class lever mechanism: *L_input_* means the length of food arm (*L_FA_*) and *L_output_* means the length of the sensor arm (*L_SA_*).

The actual weight of the food placed on the spoon is minuscule, so highly sensitive detecting sensors and systems are absolutely required. The sensing mechanism of the ring sensor is shown in Figure 2a. When the sensor is pressed, contact between the pressure-sensitive material and the electrode occurs. As the pressure increases, the contact area increases, and the total resistance decreases. This resistance change is detected through the voltage distribution principle. The overall mechanism for sensor operation is described as shown in the flowchart in Figure 2b.

We enhance the sensitivity of the sensing system with three mechanical factors: (1) a first-class lever modified mechanism; (2) a force focusing structure consisting of protrusions of the ring surface; and (3) a dual pad sensor. First, the lever effect can improve the sensitivity of the system [24,25,26]. There are three classes of levers, and among them, we use the first-class lever mechanism to enhance the sensitivity. The first-class lever is used to create a large force at the load point by applying a small force to the effort point on the other side of the fulcrum. The advantage of the general lever law is that a small force can be used at the effort point. In the case of the ring sensor, when lightweight food is placed at the effort point, a large force is applied to the ring sensor, which corresponds to the load force position of the first-class lever. Thus, we can amplify the force applied to the ring sensor via the first-class lever mechanism.

The specific details of the force amplification are shown in Figure 2c. The food mass is the input force and the force detected by the ring sensor is the output force. Here, *L_input_* is the length of the food arm (*L_FA_*), and *L_output_* is the length of the sensor arm (*L_SA_*). Also, the position where the spoon is pressed and fixed by the thumb serves as a support point. The magnitude of the force amplification is calculated by the lever equation, *F_output_ = F_input_ (L_input_/L_output_)*. This lever effect depends on where the spoon is held. We also use a dual pad sensor, and it is expected that the dual effect will be obtained by applying the dual pad sensor in the same limited area. We use a unique structure that focuses the force on the sensor node by using protrusions on the ring. On a flat surface, force is dispersed globally, so there is a loss of force at the sensor node, whereas protrusions concentrate the force on the sensor node. We anticipate that the pressing region is limited to an active area, so it generates high pressure even if the force is the same.

## 3. Results

### 3.1. Characteristic Analysis of Sensor Signals to Spoon Arm Ratios for Different Sensor Grip Positions

**Figure 3:**
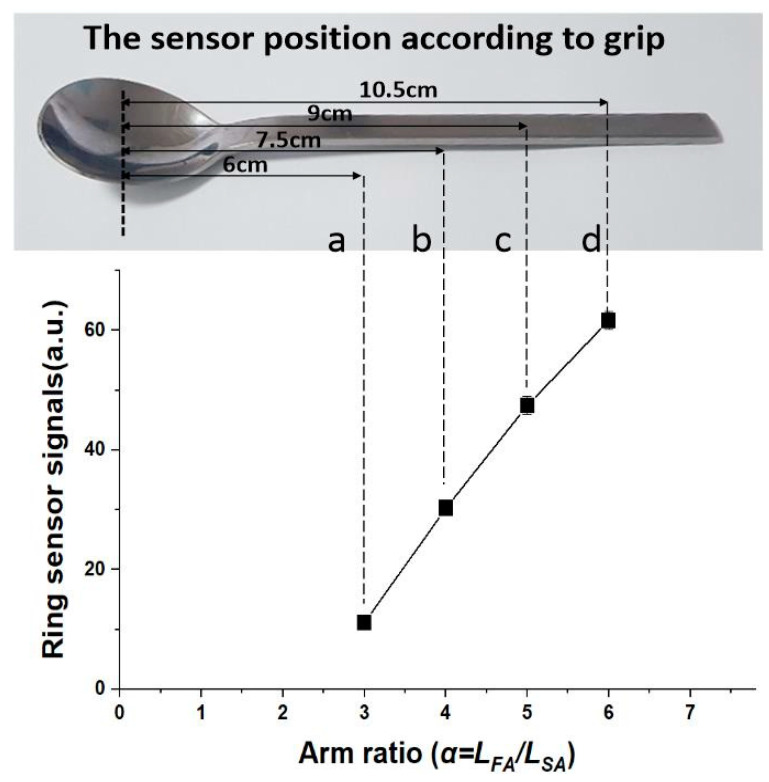
Experimental verification result of the first-class lever mechanism through ring sensor signals according to the gripped position of the spoon (*n* = 15). The sensor signal increases in proportion to the arm ratio, where the sensor position is the position of the thumb when holding the spoon and *L_SA_* is fixed at 1.5 cm.

We experimentally validated the influence of the three components that provided sensitivity enhancement. First, we verified the effects of the first-class lever mechanism. For proof-of-concept, the sensor was wound and tested as a sticker directly on the finger without the ring apparatus. Before loading food, the baseline was reset to take into account the spoon’s own weight. The distance of the food arm, which is the distance between the middle finger of fulcrum and food that acts as the input force, was set to 4.5, 6, 7.5, and 9 cm. The sensor arm’s length, which is the distance between the fulcrum and the sensor of the output force point, was fixed at 1.5 cm. The mass of food was set at 5 g. As shown in the graph of Figure 3, we confirmed that, as the food arm increased, the signal of the sensor was amplified by the first-class lever mechanism. It was confirmed that the minuscule mass of the food could be sensed through the amplified signal provided by the first-class lever mechanism using a sensing system located on the thumb. The reason the amplification was larger in the real environment than the ratio from the lever formula is that the inclination of the spoon naturally increases as the fulcrum point moves toward the ring sensor. Therefore, even with the same food mass on the spoon, the ring sensor is stimulated with a greater force when the spoon is tilted.

### 3.2. Effect of the Number of Ring Sensor Pads on Ring Sensor Signals: Single Versus Dual

**Figure 4:**
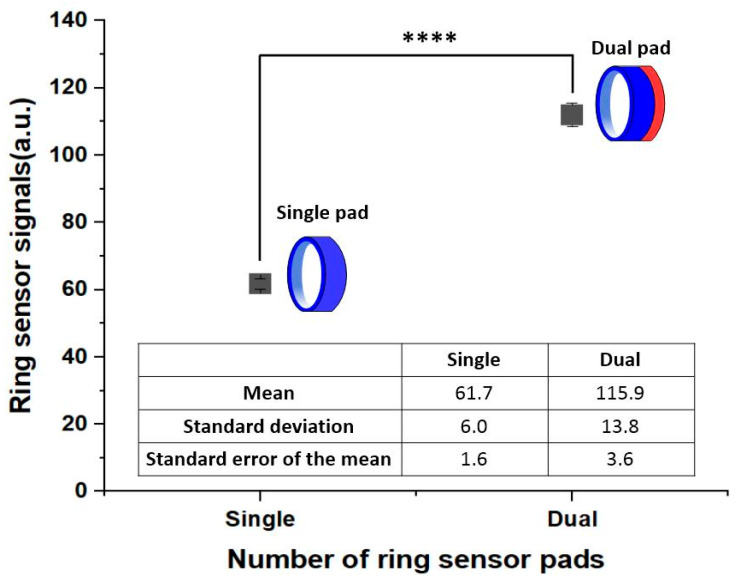
Experimental results for the ring sensor signals according to the number of sensor pads (*n =* 15). The sensor’s signal strength of the dual pad was almost twice that of the single pad. Asterisks are denoting the statistical significance, ranging from significant (*) to extremely significant (****).

Here we aimed to show the effect of the number of ring sensor pads. The sensor was overlapped and wound directly on the finger to show only pure pad number effects in the limited area. The food mass and arm ratio were set at 5 g and 6.0, respectively. The dual pad’s signal intensity was significantly different from that of the single pad (*p-*value = 2.0 × 10^–10^). We confirmed the ring sensor signal on the dual pad was 1.88 times that of the single pad, as shown in Figure 4.

### 3.3. Effect of the Ring Surface Structure on Ring Sensor Signals: Plain Versus Protrusions

**Figure 5:**
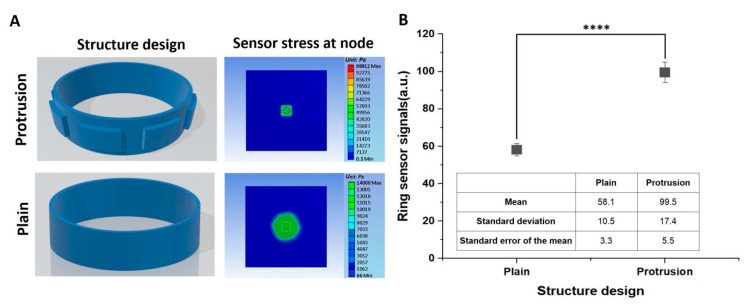
Numerical simulations and experimental results for the effect of the protrusion structure on ring sensor signals (*n* = 10). (**A**) Analysis of the finite element simulation of the protrusion and plain structures, where the white dashed line denotes the size of the protrusion. (**B**) The effect of the protrusion structure in comparison to the planar surface, with a *p-*value of 1.5 × 10^–5^.

We also confirmed the effect of protrusions relative to a flat structure. First, as shown in Figure 5A we simulated the protrusion effect with finite element analysis using the Ansys mechanical tool (Canonsburg, PA, USA). The simulations suggested a 1.88 times performance increase of the protrusion structure compared to the flat structure. To verify this, we experimentally compared the protrusion structure and planar structure on the ring apparatus. The protrusion structure on the ring surface consisted of seven protrusion arrays that were 5 × 4 mm^2^ in size with an array pitch of 7.45 mm, which is the pitch of the sensor node. The protrusion size was set based on force propagation and the sensor node size (5 × 6 mm^2^).

The food mass and arm ratio were set at 10 g and 6.0, respectively. As shown in Figure 5B, the sensor’s signal strength of the protrusion and plain structure was significantly different, with a *p-*value of 1.5 × 10^–5^. The sensitivity of the protrusion structure was 1.71 times that of the flat structure. The increased sensitivity was caused by protrusions locally concentrating the force on the sensor node.

### 3.4. Ring Sensor’s Performance Analysis for Food Detection Using the F_1_ Score

We checked the classification results on the eating behavior using a confusion matrix to grasp the ring sensor system’s performance. For the test, we used a mass of 5 g. Accuracy was computed as the following Equation (1), and the F_1_ score was calculated using a harmonic mean of Precision and Recall from Equations (2)–(4).

*Accuracy* = *(TP* + *TN*)/(*TP* + *FN* + *TN* + *FP*) (1)

*Precision* = *TP*/(*TP* + *FP*) (2)

*Recall* = *TP*/(*TP* + *FN*) (3)

(4) $$F_{1}\;=\;(2\;\mathrm{Precision}\;\cdot \;\mathrm{Recall})/(\mathrm{Precision}\;+\;\mathrm{Recall})$$

where TP (true positive) means that when there was a 5 g mass on the spoon, a signal above the threshold was generated, and the FP (false positive) was defined as when a signal above the threshold was generated when there was no mass on the spoon. The experiment was conducted by dividing it into a vertical movement and a circular movement on a plane, a typical case where the spoon moves without food. As shown in Table 1, an accuracy of 0.963 and an *F*_1_ score of 0.962 were calculated for vertical movement. Accuracy of 0.983 and an *F*_1_ score of 0.983 were obtained for circular motion on a plane. In the case of food with vertical movement, the rate of misrecognition, that is, the frequency in which actual food was present but not detected was relatively high. It was determined that in case of vertical motion without mass on the spoon, the spoon’s weight stimulates the sensor of the finger when the vertical motion stops, due to effect of acceleration. For this reason, an error occurred even when there was no mass on the spoon.

### 3.5. Correlation Analysis between Food Mass and Signal of the Ring Sensor

**Figure 6:**
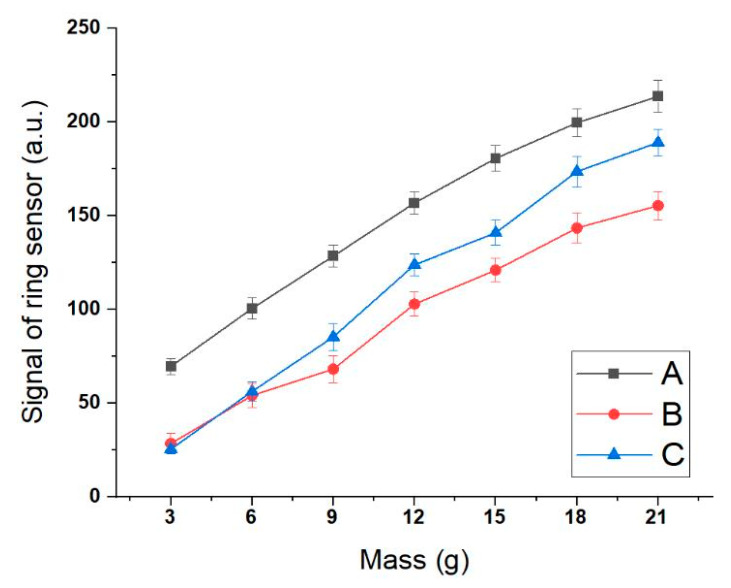
Correlation between the sensor signal and food mass on spoon using a ring sensor system (*n* = 15). The experiment was conducted by three users, and ring sensor signals increase proportionally according to the mass placed on the spoon. For A, B, and C the *R*^2^ values were 0.986, 0.989, and 0.990, respectively. The spoon arm ratio was set to 6.0. The error bar indicates the standard error of the mean.

We confirmed the device’s accuracy with the dual-padded sensor and the protrusion structures using a correlation of the ring sensor signal and the food mass. When masses of 3, 6, 9, 12, 15, 18, or 21 g were placed on a spoon, the corresponding analog signal of the sensor was measured. The experiment was performed for three users, with the results shown in the graph in Figure 6. We believe that the difference between the graphs occurred due to the sensors’ deviation and the users’ proficiency. However, the *R*^2^ values were very high, with an average value of 0.988. Finally, we verified the detection of a signal proportional to the mass of food on a spoon and showed the feasibility of a personalized digital medicine solution capable of quantitative intake sensing.

## 4. Discussion

### 4.1. Supplementary Needs of the Ring Sensor System as a Food Mass and Intake Detector

A few complementary additions could add to the quality of the ring sensor as a useful healthcare application for real-life dietary monitoring. In the protrusion structure experiment, complete contact between the spoon and the ring sensor was not possible due to the ring’s thickness and the ring’s and spoon’s rigidity. Therefore, if the modulus of the ring material was tuned, it is expected that improved results could be obtained. Also, in the current system, it is necessary to locate the finger grip position manually. Therefore, it is also necessary to insert the touch sensor so that the position of the spoon grip can be automatically known. Through the finger’s gripping position, the signal intensity as it is changed by the lever law could be calculated to determine the actual FMI. Furthermore, in the future it will be necessary to determine the amount of food when using different tools, such as forks and chopsticks.

### 4.2. Synergy Effect of Combining the Ring Sensor with Different Fields

The ring sensor system has the attractive ability to measure accurately the mass of food consumed with a spoon. To achieve the ultimate goal of dietary monitoring, a hybrid approach is required that will combine different fields beyond the mixture of sensors to identify the types and mass of foods ingested. For example, in the case of liquid and solid foods, a sensing error can occur due to the difference in the center of mass when placed on a spoon even when the weight is the same. This difference can be compensated by merging the spoon sensor with smart glasses to analyze intake patterns such as chewing and drinking [24,25,26]. The combination with smart glasses would make it possible to reduce the error through mass calibration based on the spoon shape and the center of gravity by determining whether the form of the ingested food is solid or liquid. Smart glasses will also be useful for continuous monitoring. Using smart glasses makes it possible to clearly distinguish whether or not the actual food is ingested. Through this, it is possible to continuously monitor the intake amount through an automatic baseline adjustment and calculation process when food intake is completed.

## 5. Conclusions

The essence of this study is that it provided a direct and objective measurement method for FMI. Moreover, we developed a highly sensitive dual-padded ring-type sensor with a protrusion structure to detect and monitor FMI. The improved sensitivity of the system was based in part on its use of a lever mechanism. The sensitivity could be increased by 1.88 and 1.71 times by using a double layer and protrusion structures, respectively. The sensor accuracy shows a high correlation of ring sensor signal to food mass, with *R*^2^ = 0.988 and an average *F*_1_ score of 0.973.

The previous method only determined the intake pattern and the amount of chewing, so there was a limit to the practical dietary information that could be obtained. However, we demonstrated the feasibility of FMI analysis using a ring-type bio-sensing system for actual dietary monitoring. Therefore, we believe that this approach is potentially beneficial for developing a wearable platform to support the prevention of obesity, which causes a number of adult diseases. Furthermore, metabolic syndrome can be prevented, and physical activity can be revitalized through dietary monitoring. Therefore, a dietary monitoring system using a ring sensor is expected to be useful as a diagnostic indicator that identifies physiological signals based on the individual’s dietary behavior.

## Figures and Tables

**Table 1:** Confusion matrix for accuracy and *F*_1_ score analysis of food detection.

Movement Categories		Actual Activities	
		Food on Spoon	No Food on Spoon
Category I (Vertical movement)	Food on spoon	141	2
	No food on spoon	9	148
	Accuracy	0.963	
	*F*_1_ score	0.962	
Category II (Circular movement on the plane)	Food on spoon	148	3
	No food on spoon	2	147
	Accuracy	0.983	
	*F*_1_ score	0.983	

## Nomenclature

FMI	Food mass and intake
EGG	Electroglottography
PET	Polyethylene terephthalate
PC	Polycarbonate
PMMA	Poly(methyl methacrylate)
PSA	Pressure-sensitive adhesive
FPGA	Field-programmable gate array
L_FA_	Length of the food arm
L_SA_	Length of the sensor arm
TP	True positive
FN	False negatives
FP	False positive
TN	True negative
